# Invasive Process and Repeated Cross-Sectional Surveys of the Mosquito *Aedes japonicus japonicus* Establishment in Belgium

**DOI:** 10.1371/journal.pone.0089358

**Published:** 2014-04-02

**Authors:** David Damiens, Audrey Ayrinhac, Wim Van Bortel, Veerle Versteirt, Wouter Dekoninck, Thierry Hance

**Affiliations:** 1 Université catholique de Louvain, Earth and Life Institute, Biodiversity Research Centre, Louvain-la-Neuve, Belgium; 2 Insect Pest Control Laboratory, International Atomic Energy Agency, Vienna, Austria; 3 Institute of Tropical Medicine, Dept. Parasitology, Antwerpen, Belgium; 4 Avia-GIS, Precision Pest Management Unit, Zoersel, Belgium; 5 Royal Belgian Institute of Natural Sciences, KBIN-IRSNB, Brussels, Belgium; University of Tours, France

## Abstract

When accidentally introduced in a new location, a species does not necessarily readily become invasive, but it usually needs several years to adapt to its new environment. In 2009, a national mosquito survey (MODIRISK) reported the introduction and possible establishment of an invasive mosquito species, *Aedes j. japonicus*, in Belgium. First collected in 2002 in the village of Natoye from a second-hand tire company, then sampled in 2003 and 2004, the presence of adults and larvae was confirmed in 2007 and 2008. A repeated cross-sectional survey of *Ae. j. japonicus* was then conducted in 2009 in Natoye to study the phenology of the species on two different sites using three kinds of traps: Mosquito Magnet Liberty Plus traps, BG sentinel traps and CDC Gravid traps. An analysis of the blood meals was done on females to assess the epidemiological risks. Five species of mosquitos were caught using the different kind of traps: *Culex pipiens, Cx torrentium, Anopheles claviger, Aedes geniculatus and Ae. j. japonicus*, *Cx pipiens* being the most abundant. The CDC gravid traps gave the best results. Surprisingly *Ae. j. japonicus* was only found on one site although both sites seem similar and are only distant of 2.5 km. Its population peak was reached in July. Most of the engorged mosquitoes tested acquired blood meals from humans (60%). No avian blood meals were unambiguously identified. Larvae were also collected, mostly from tires but also from buckets and from one tree hole. Only one larva was found in a puddle at 100 m of the tire storage. A first local treatment of *Ae. j. japonicus* larvae population was done in May 2012 using *Bacillus thuringiensis* subsp. *israelensis* (*Bti*) and was followed by preventive actions and public information. A monitoring is also presently implemented.

## Introduction

The process of invasion by exotic species follows at least six well identified steps: 1) transport, 2) introduction, 3) colonization, 4) naturalization, 5) spread and 6) impact [1]. Notwithstanding this classification was first developed for plants, it is also useful for animals, particularly when they may become agricultural, medical or veterinary pests. This categorization can be limited to 3 main stages: initial dispersal, establishment of self-sustaining populations, and spread [2]. They point out the lack of data on stage 1 citing that only 6.2% of the papers they analysed contained empirical data, although stage 1 is the best stage to act with direct management strategies [2]. In 2007, a Belgian national mosquito survey (MODIRISK) was launched to study the taxonomic and functional biodiversity of both endemic and invasive mosquito species. The first phase of this inventory was based on a random selection of 1000 collection points in three main environmental categories: urban, rural, and natural areas. During that survey the invasive *Aedes (Finlaya) japonicus japonicus* [3] (*Ochlerotatus japonicus japonicus sensus* [4] and *Hulecoetomyia japonica japonica* sensus [5]) was found at two locations only, in the same village of Natoye [6], [7]. The native range of *Ae. j. japonicus* covers Northeast Asia (Japan, Korea, South China) to the far east of Russia. *Ae. j. japonicus* was also simultaneously detected in Connecticut in 1998 [8] in the states of New York and New Jersey [9]. *Ae. j. japonicus* has now expanded to 22 American states and to parts of Canada [10]. In Europe, some larvae were identified in one location in France and rapidly eradicated [11], [12]. A survey of 3548 potential sites in Switzerland in 2008 showed that *Ae. j. japonicus* has spread over an area of 1400 km^2^ including the border of Germany [12]. The species is now considered to be widely spread in central Europe [13]. Updated information on its distribution can be found on the ECDC's website: http://ecdc.europa.eu/en/healthtopics/vectors/vector-maps/Pages/VBORNET_maps.aspx. In Belgium, larvae of *Ae. j. japonicus* were first identified in October 2002 on the premises of a second-hand tire company in the village of Natoye (24 km SE Namur) [6]. New specimens have been then sampled using different methods in two consecutive years (2003–2004). Finally, in 2007 and 2008, its presence as adults and larvae was confirmed based on morphological and molecular identification [6]. In spite of its, at least, 8-year presence, *Ae. j. japonicus* still seems to be in either the introduction [1] or initial dispersal phase [2].

In its native range *Ae. j. japonicus* prefers to oviposit in rock holes [14]. However, in Belgium, larval collections revealed preferences for artificial sites, mostly tires with water and debris as in most countries where the species has been found [8], [15], [16]. Mosquito host preference can vary by location [16]. Females are known to feed on mammals, including humans, in the field [17], [18], and on avian hosts under laboratory conditions [20], [21], [22], and could therefore act as a zoonotic bridge vector species. Under laboratory conditions, this mosquito has been shown to be a competent vector of Eastern encephalitis virus, La Crosse virus, St. Louis encephalitis virus, and a highly competent vector for West Nile virus [23], [24], [25], [26], [21]. However, its role as a disease vector species under natural conditions in the United States, where the species has been established for almost a decade, remains unclear.

Because of health risks, developing an efficient control program for *Ae. j. japonicus* is essential. The successful elaboration of such a program requires high quality information about the onsite population dynamics of the species. A repeated cross-sectional survey of *Ae. j. japonicus* larvae and adults was thus conducted in 2009 in Natoye to determine the phenology of the species, the peak of activity, the number of and the kind of larval habitats used and its relationships with the presence of other mosquitos species of the native mosquitos community. Moreover, determination of host-feeding preferences was done to assess the potential epidemiological risks caused by the presence of *Ae. j. japonicus*.

## Materials and Methods

### Study sites

Two Belgian second-hand tire companies located in the village of Natoye (Namur) were surveyed. Sites were named Natoye 1 (50.3389587°N, 5.044879°E) and Natoye 2 (50.33588°N, 5.0714698°E) and are about 2.5 km away as the crow flies. We thank the owners of the tire companies for their collaboration and goodwill to sustain the many visits on their property. The companies import tires from other European countries, mainly for trucks and heavy vehicles. Tires are stacked outside where many are exposed to rainfall and may contain water and organic material such as decomposing leaves. Land cover consists largely of town, deciduous forests, gardens, cultivated field around Natoye 1 and gardens, cultivated fields and meadows around Natoye 2.

### Adult survey

**Figure 1:**
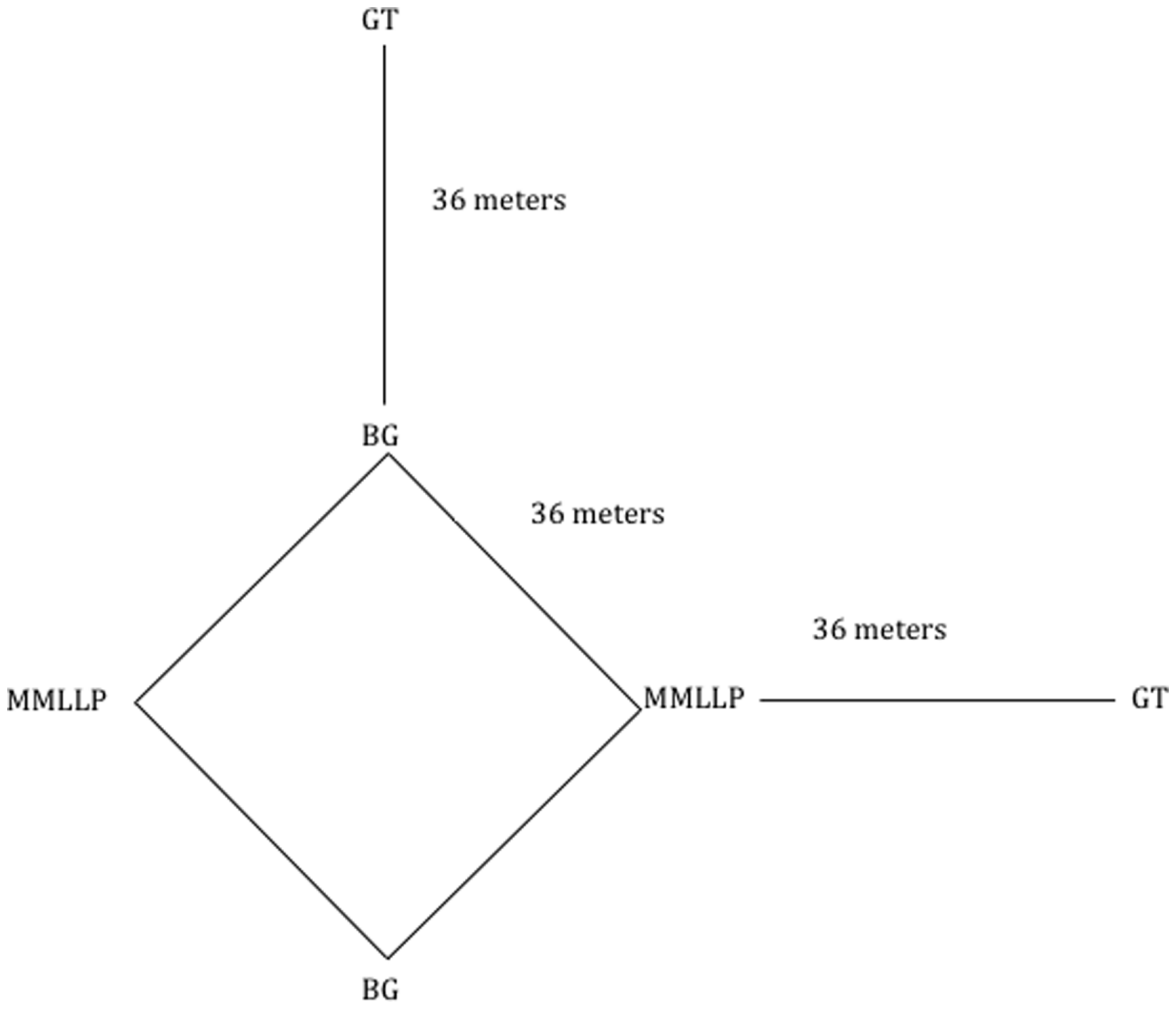
Position of traps to minimize interference Mosquito Magnet Liberty plus (MMLP), BG sentinel (BG) and gravid traps (GT).

The sites were sampled 13 times, every two weeks from the 20th of April 2009 until the 5th of October 2009. For each sampling three types of traps were used during two consecutive days (12:00 am–12:00 am). The different traps used were Mosquito Magnet Liberty Plus traps (Woodstream Corporation, Lititz, PA), BG sentinel traps containing the BG-Lure® attractant (Biogents AG, Regensburg, Germany) and CDC Gravid traps (model 1712, John W. Hock Co., Gainesville, FL). The water used in CDC traps was obtained from a mixture of fresh grass clippings or hay and brewers yeasts fermenting in water during 5–7 days [27]. Each site was divided in two sub-sites. Each sub-site has been sampled with one trap of each type (i.e. 3 different traps per sub-site, 6 traps per site). Traps were placed in a way to reduce interference between traps to a minimum (Figure 1). Although the three types of traps are mainly attractive to females, both males and females trapped were collected and identified.

### Larval survey

Sampling for immature mosquitoes was conducted on the two Natoye sites during four visits in May, August, September and October 2009. During each visit, an extensive inventory of potential larval habitats (all water-holding sites) coupled to a search for mosquito immature stages was done on company grounds and within a 500 meter perimeter of the tire stock. At each visit, about 20 potential larval habitats such as tires and artificial containers and abandoned buckets were screened on the company location and in the immediate vicinity. *Ae. j. japonicus* larvae were collected from potential larval habitats by using a pipette and white plastic bowl, and transported alive to the laboratory in vials labelled with larval habitat specific identification details. In the lab, the larvae were first transferred with a dropper to a small cup or bowl with fresh clean water as a washing procedure. The procedure was repeated until the total elimination of debris or sediment. To avoid larval collapse, larvae were then killed by thermal shock with hot water (60°C), collected with a fine pipette and put in a vial filled with a 80% solution of ethanol. Treated larvae were then identified using a stereoscopic microscope. Pupae were reared to adult in a secured lab for identification.

### Morphological Identification

Mosquito larvae and adults were identified using identification keys [20], [28], [29], [30].

### Blood meals identification

Fifty-four adult females of *Ae. j. japonicus*, captured in gravid traps in Natoye during the 2009 campaign, were immediately frozen at −80°C after identification. The abdomens were then dissected with sterile pincels and DNA was extracted with salt/chloroform extraction protocol [31]. The concentration and purity were determined from the A_260_/A_280_ ratio using a spectrophotometer. The concentration of all samples was then homogenized at 50 ng/µl.

Presence of host blood was determined by polymerase chain reactions (PCR), and amplified products were visualized on 2% agarose gel (see Table 1 for primer pairs). DNA of each sample was analysed for the presence of mammal blood using a primer pair, specific to a region of the cytochrome b mitochondrial gene (Cyt b) [32]. Samples positive to the presence of mammalian blood were then subjected to a multiplex PCR to discriminate among dog, human, cow and sheep/goat blood [33]. Presence of avian blood was tested with a primer pair that specifically amplifies the NADH dehydrogenase subunit 2 (ND2) mitochondrial gene [34]. Undetermined mammals and avian products were sequenced using a 377 DNA analyzer (Applied Biosystems). Multiplex PCR reactions were performed using Qiagen Multiplex PCR kit.

**Table 1:** Primer sequences used for the blood meal identification assay.

PCR	primer name	primer sequence	size	Source
Mammal	MAMMAL-For	CGAAGCTTGATATGAAAAACCATCGTTG	772	[32]
	MAMMAL-Rev	TGTAGTTRTCWGGGTCHCCTA		
Avian	L5216-For	GGCCCATACCCCGRAAATG	1041	[34]
	H6313-Rev	ACTCTTRTTTAAGGCTTTGAAGGC		
Multiplex mammals	Human741F	GGCTTACTTCTCTTCATTCTCTCCT	334	[33]
	Goat894F	CCTAATCTTAGTACTTGTACCCTTCCTC	132	
	Cow121F	CATCGGCACAAATTTAGTCG	561	
	Dog368F	GGAATTGTACTATTATTCGCAACCAT	680	
	UNREV1025	GGTTGTCCTCCAATTCATGTTA	-	

## Results

### Adult survey

**Figure 2:**
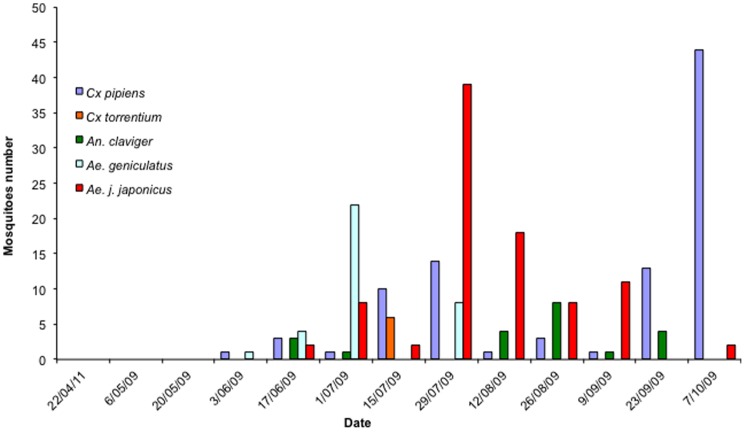
Number of adult mosquitoes of the 5 species found in the three kinds of traps during the 13 visits at Natoye 1.

**Figure 3:**
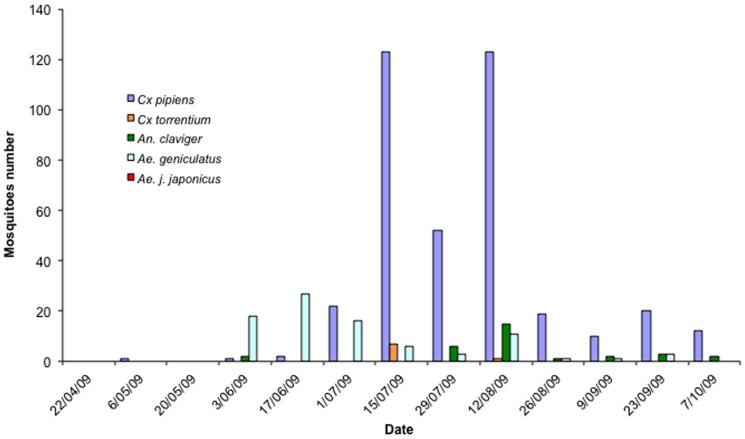
Number of adult mosquitoes of the 4 species found in the three kinds of traps during the 13 visits at Natoye 2.

During the sampling season, 753 adult mosquitoes were collected from both sites belonging to five species for Natoye 1 and four species for Natoye 2. The greatest numbers of mosquitoes were collected in the CDC Gravid traps (473 mosquitoes) while 214 and 66 mosquitoes were found respectively in the Mosquito Magnet Liberty Plus traps and in the BG sentinel traps. At both sites, the most abundant species was *Culex* (*Culex*) *pipiens* L. s.l. (63.2%). The species *Aedes* (*Finlaya*) *geniculatus* (Olivier) (16.1%), *Anopheles* (*Anopheles*) *claviger* (Meigen) (6.9%) and *Culex* (*Culex*) *torrentium* (Martini) (1.9%) were also caught. *Ae. j. japonicus* individuals were found only at Natoye 1 (11.9% of the number of sampled individuals) (Figures 2 and 3). All individual were females, excepted 18 males of *Ae. geniculatus* and 2 males of *C. pipiens* on respectively 115 individuals and 476 individuals. Table 2 shows the distribution of mosquito species per type of trap and per site. Of the 243 mosquitoes collected in Natoye 1, 90 were *Ae. j. japonicus* (37%). *Ae. j. japonicus* was first recorded at Natoye 1 in mid-June. The population increased then from June to August and decreased until October. *Ae. j. japonicus* seemed to be a ‘late season’ species while *Ae. geniculatus* appeared earlier in the season but disappeared rapidly at the end of July.

**Table 2:** Total numbers and percentages of adult mosquitoes of the 5 species found per trap kind during the 13 visits at Natoye 1 and Natoye 2.

		*Cx. pipiens*	*Cx. torrentium*	*An. claviger*	*Ae. geniculatus*	*Ae. japonicus*
Natoye 1	M	58	0	19	23	2
	B	8	0	2	1	1
	G	25	6	0	11	87
	T	91	6	21	35	90
	%	37.5	2.5	8.6	14.4	37.0
Natoye 2	M	20	0	15	77	0
	B	35	0	15	3	0
	G	330	8	1	6	0
	T	385	8	31	86	0
	%	75.4	1.6	6.1	16.9	0

M = Mosquito Magnet Liberty Plus traps, B = BG sentinel traps, G = Gravid traps, T = Total, % are calculated per site.

### Larval survey

From the four visits, a total of 71 and 65 larval habitats with larvae were identified in Natoye 1 and Natoye 2 respectively. In Natoye 1, a total of 786 larvae belonging to 10 species were collected and 922 larvae belonging to 7 species were collected in Natoye 2, from which 784 and 909 individuals were identified at the species level respectively (Table 3). The first observation of *Ae. j. japonicus* larvae was in May. A total of 323 *Ae. j. japonicus* larvae were collected only around Natoye 1, the majority of which were recovered from tires (138 larvae; 42.7%) and other artificial sites such as buckets on company grounds (143 larvae, 44.3%). Although less used, natural sites, such as one tree hole (25 meters from company) also harboured notable numbers of larvae (41 larvae, 12.7%). Finally, one larva was found in a puddle (about 100 meters from the company). *Cx. pipiens* larvae were also found in 25 sites of the 32 sites where *Ae. j. japonicus* larvae were present. *Cx. torrentium* were recorded in 3 sites of those 32. All larval stages were mixed in these two cases.

**Table 3:** Total number of larvae of each species caught in Natoye 1 (71 larval habitats) and Natoye 2 (65 larval habitats).

	*Cx. pipiens*	*Cx. torrentium*	*An. claviger*	*Ae. geniculatus*	*Ae. j. japonicus*
Natoye 1	416	31	4	10	323
Natoye 2	838	26	1	44	0

### Blood meals identification

From the 54 female mosquitoes included in the analysis, 25 contained a detectable amount of a blood meal. A majority of them were single meals (23 mosquitoes, 92%), and only two mosquitoes (8%) were positive for two host species. Analysis of the blood-meal sources identified mammals as the only source of blood. Humans were the most frequent mammalian blood meal source (60%) followed by cows (32%) and 2% of mixed origin (human and cow) (Table 4). Four samples were positive for the amplification of the avian fragment but the sequencing did not give any results. As we cannot be sure of these results, these positive samples for avian origin can unfortunately not be taken into account.

**Table 4:** Blood meal identifications from *Ae. j. japonicas*.

	Human	Cow	Human and Cow
Nb of blood-meal identified	15	8	2
%	60	32	8

## Discussion

With this follow up study, we confirmed the presence of *Ae. j. japonicus* in Belgium [5]. According to invasive classifications [1], [2] and considering that larvae were identified in natural sites outside the tires, *Ae. j. japonicus* is probably in the colonization or establishment of self-sustaining populations phase at least in Natoye 1 in Belgium. In 2009, this exotic species was only found in Natoye 1 whereas it was found at both Natoye sites in past years [5]. In Natoye 1, the population presented its maximum abundance in July (39 females only caught in Gravid traps). This peak of abundance of *Ae. j. japonicus* females corresponds to that reported for Connecticut [7] and Ontario in Canada where the species is spreading [35] with the majority of specimens collected in July, August and September.

In Natoye 1, tires of different dimensions (car to truck tires) were stored outside waiting for retreatment. They constituted artificial pools of fresh water with leaf remains (presence of a nearby forest) and underwent frequent partial emptying and refilling, creating ideal sites for *Ae. j. japonicus* oviposition and hatching. In these artificial larval habitats, *Ae. j. japonicus* larvae were frequently found with *Cx. pipiens* larvae. This association is common and was also found in Canada [34] and in the United States [7], [36]. However, in these entomological surveys, *Ae. j. japonicus* larvae were associated with numerous other species including *Culex restuans* Theobald, *Aedes triseriatus* (Say) and *Aedes atropalpus* (Coquillett). It is interesting to note that presence of *Ae. j. japonicus* and *Cx. pipiens* larvae within the same larval habitats in Natoye 1 poses the question of a coexistence of both species and possible larval competition. For instance, *Cx. pipiens* in Natoye 1 shows an abundance reduced by a half compared to its abundance in Natoye 2. This decline in the relative abundance of *Cx. pipiens* appears to be concomitant with the abundance peak of *Ae. j. japonicus* suggesting a negative effect of the presence of the invasive species on the native species population. The same trend was observed for *Ae geniculatus* that disappeared in Natoye1 after the peak of *Ae. j. japonicus* whereas it remained present at the same date in the other location in absence of *Ae. j. japonicus*. Similar reduction of populations of native species due to *Ae. j. japonicus* have been already documented in USA [15].

The absence of *Ae. j. japonicus* in Natoye 2 may be due to several reasons ranging from human, climatic and ecological determinants such as a greater tires turn-over than in Natoye 1 associated with an absence of natural oviposition sites (no forest near the company contrarily to Natoye1). Indeed, in Natoye 1, larvae have been collected from a variety of artificial habitats which are the most abundant container types present on the site but also from natural habitat (frequently in a tree hole and one occasion in a puddle in the forest). These natural larval habitats possibly serve as “refuge” for *Ae. j. japonicus* and allow species to stay on the sites over the years in spite of the rapid turn-over of tires (a part of the tires is removed every 3 months). Moreover, its presence since 2002 and the recording of larvae in early May indicate that this species has probably overwintered under the Belgian weather conditions. In the future, studies on the overwintering capacities and the genetic structure of the population are needed to determine if the population survives throughout the year or if there are multiple introductions each year through tire importation.

It appeared that gravid traps are the most effective for collecting *Ae. j. japonicus* adults (87 females caught, Table 2) as compared to the Mosquitoes magnet trap (2 captures) and the BG sentinel trap (1 capture). In Natoye 2, *Cx. pipiens* was also more captured in Gravid Trap (330 females) than for the other kind of traps whereas, surprisingly more individual were caught in the Mosquito magnet traps in Natoye 1 where *Ae. j. japonicus* was also present. Gravid trap are a good indicator of the abundance of females searching for an oviposition site whereas the two other kinds of traps are linked to the blood-meal searching process and may be less effective for some species. In Canada [35] and in United States [27], a higher proportion of specimens were collected using gravid traps compared to light traps. The least effective traps appear to be the BG-Sentinel containing the mosquito attractant BG-Lure®. This attractant releases a combination of non-toxic substances found on human skin (lactic acid, ammonia, and fatty acids) and has been proven to be a reliable and standardized tool for collecting *Aedes aegypti L.* in urban areas [37], *Aedes albopictus* and other anthropophagic mosquitoes. However, in our study, the BG-Sentinel traps showed a low efficiency for monitoring *Ae. j. japonicus*.

Our results on blood-meal origins in the Belgian population of *Ae. j. japonicus* are consistent with previous observations made in the US where mammals are the principal hosts. The study of *Ae. j. japonicus* host-preferences in the Northeastern USA [18], [38], [19] has shown an exclusive feeding on mammalian blood, mostly on deer. Interestingly, human is the most preferred host of the Belgian population whereas it was a secondary host in the US population [38], [19]. Even if this species can feed on quails in laboratory conditions [22], we did not find clear evidence for avian host feeding. During the blood meal analysis, four samples showed amplification of the avian primer pair, but as the sequencing of the amplified fragments failed, we are unable to confirm that result. The non-exclusive preference for Belgian *Ae. j. japonicus* population to feed on humans highlights the epidemiological risks generally associated with this species, as it could potentially serve as a bridge vector for the transmission of flaviviruses like West Nile virus between mammals [39].

This repeated cross-sectional survey addressed questions relevant to the development of a pest risk assessment of the exotic species *Aedes j. japonicus* and offers assistance in administrative decision-making. To date, it seems that *Ae. j. japonicus* is well-established in the site Natoye 1. However, there are currently no indications that the species, which has been present for at least six years in Belgium, has spread to the surroundings. However, larger infestations cannot be completely ruled out. An adapted treatment needed to be developed based on our results and therefor the competent authorities have been warned. The most frequently used oviposition sites of *Ae. j. japonicus* were tires and buckets located on the company site.

Following these observations, an action plan was developed. A first local treatment of *Ae. j. japonicus* larvae population was done in May 2012 using sprayings of VectoBac® WG and applications of Vectomax® G granules (*Bacillus thuringiensis* subsp. *israelensis*). It was followed by a monthly monitoring using gravid traps and larval habitat observations. Moreover, preventive actions were also implemented to reduce larval populations, by emptying the tires present on site and sheltering them from rainfall and by an appropriate information of the population (http://biodiversite.wallonie.be/fr/le-moustique-japonais.html?IDC=5667).
